# Expression quantitative trait methylation analysis elucidates gene regulatory effects of DNA methylation: the Framingham Heart Study

**DOI:** 10.1038/s41598-023-39936-3

**Published:** 2023-08-10

**Authors:** Amena Keshawarz, Helena Bui, Roby Joehanes, Jiantao Ma, Chunyu Liu, Tianxiao Huan, Shih-Jen Hwang, Brandon Tejada, Meera Sooda, Paul Courchesne, Peter J. Munson, Cumhur Y. Demirkale, Chen Yao, Nancy L. Heard-Costa, Achilleas N. Pitsillides, Honghuang Lin, Ching-Ti Liu, Yuxuan Wang, Gina M. Peloso, Jessica Lundin, Jeffrey Haessler, Zhaohui Du, Michael Cho, Craig P. Hersh, Peter Castaldi, Laura M. Raffield, Jia Wen, Yun Li, Alexander P. Reiner, Mike Feolo, Nataliya Sharopova, Ramachandran S. Vasan, Dawn L. DeMeo, April P. Carson, Charles Kooperberg, Daniel Levy

**Affiliations:** 1 Framingham Heart Study, Framingham, MA USA; 2 Population Sciences Branch, Division of Intramural Research, National Heart, Lung, and Blood Institute, National Institutes of Health, Bethesda, MD USA; 3 Friedman School of Nutrition Science and Policy, Tufts University, Boston, MA USA; 4 Department of Biostatistics, Boston University School of Public Health, Boston, MA USA; 5 Department of Ophthalmology and Visual Sciences, University of Massachusetts Chan Medical School, Worcester, MA USA; 6 Mathematical and Statistical Computing Laboratory, Office of Intramural Research, Center for Information Technology, National Institutes of Health, Bethesda, MD USA; 7 Department of Neurology, Boston University School of Medicine, Boston, MA USA; 8 Division of Clinical Informatics, Department of Medicine, University of Massachusetts Chan Medical School, Worcester, MA USA; 9 Fred Hutchinson Cancer Center, Seattle, WA USA; 10 Channing Division of Network Medicine, Brigham and Women’s Hospital, Harvard Medical School, Boston, MA USA; 11 Division of Pulmonary and Critical Care Medicine, Brigham and Women’s Hospital, Harvard Medical School, Boston, MA USA; 12 General Medicine and Primary Care, Brigham and Women’s Hospital, Harvard Medical School, Boston, MA USA; 13 Department of Genetics, University of North Carolina at Chapel Hill, Chapel Hill, NC USA; 14 Department of Biostatistics, University of North Carolina at Chapel Hill, Chapel Hill, NC USA; 15 Department of Epidemiology, University of Washington, Seattle, WA USA; 16 National Center for Biotechnology Information, National Institutes of Health, Bethesda, MD USA; 17 Department of Medicine, Preventive Medicine and Epidemiology, Boston University School of Medicine, Boston, MA USA; 18 Department of Medicine, University of Mississippi Medical Center, Jackson, MS USA

**Keywords:** Epigenetics, Transcriptomics

## Abstract

Expression quantitative trait methylation (eQTM) analysis identifies DNA CpG sites at which methylation is associated with gene expression. The present study describes an eQTM resource of CpG-transcript pairs derived from whole blood DNA methylation and RNA sequencing gene expression data in 2115 Framingham Heart Study participants. We identified 70,047 significant *cis* CpG-transcript pairs at *p* < 1E−7 where the top most significant eGenes (i.e., gene transcripts associated with a CpG) were enriched in biological pathways related to cell signaling, and for 1208 clinical traits (enrichment false discovery rate [FDR] ≤ 0.05). We also identified 246,667 significant *trans* CpG-transcript pairs at *p* < 1E−14 where the top most significant eGenes were enriched in biological pathways related to activation of the immune response, and for 1191 clinical traits (enrichment FDR ≤ 0.05). Independent and external replication of the top 1000 significant *cis* and *trans* CpG-transcript pairs was completed in the Women’s Health Initiative and Jackson Heart Study cohorts. Using significant *cis* CpG-transcript pairs, we identified significant mediation of the association between CpG sites and cardiometabolic traits through gene expression and identified shared genetic regulation between CpGs and transcripts associated with cardiometabolic traits. In conclusion, we developed a robust and powerful resource of whole blood eQTM CpG-transcript pairs that can help inform future functional studies that seek to understand the molecular basis of disease.

## Introduction

DNA methylation is an epigenetic modification characterized by the transfer of a methyl group onto DNA cytosine-phosphate-guanine (CpG) sites that can regulate gene expression. The extent of DNA methylation at specific CpG sites is associated with phenotypic variation in numerous traits including cardiovascular disease-related traits^1^ such as body mass index (BMI)^2^, blood lipids^3^, glycemic traits^4^, blood pressure^5^, and inflammatory biomarkers^6^. Expression quantitative trait methylation (eQTM) analysis identifies CpG sites that display methylation-related associations with expression of nearby (*cis*) or remote (*trans*) genes. While prior eQTM studies using commercial arrays for gene expression profiling have revealed associations between DNA methylation and gene expression that are linked to clinical disease, many of these studies are limited by small sample size and focus on specific disease phenotypes^1,7–9^.

To generate a resource of eQTM CpG-transcript pairs, we use array-based DNA methylation data and RNA sequencing (RNA-seq) gene expression data in over 2000 Framingham Heart Study (FHS) participants to precisely examine the association between whole blood DNA methylation and gene expression. We posit that leveraging RNA-seq will increase the power, precision, and relevance of our eQTM analyses beyond what was possible with previous array-based gene expression studies^7,10^. Use of RNA-seq gene expression data also provides an opportunity to interrogate DNA methylation in relation to long non-coding RNAs (lncRNAs) and explore their possible contributions to clinical disease^11,12^.

The primary aims of this study were to test the associations of DNA methylation with genome-wide gene expression and to create a resource of whole blood eQTM CpG-transcript pairs to facilitate new insights into disease pathways and processes. As proof of principle, we evaluated the associations of eQTM CpG-transcript pairs with cardiovascular traits and provide examples of how these eQTM resources can be used in future research.

## Results

**Figure 1:**
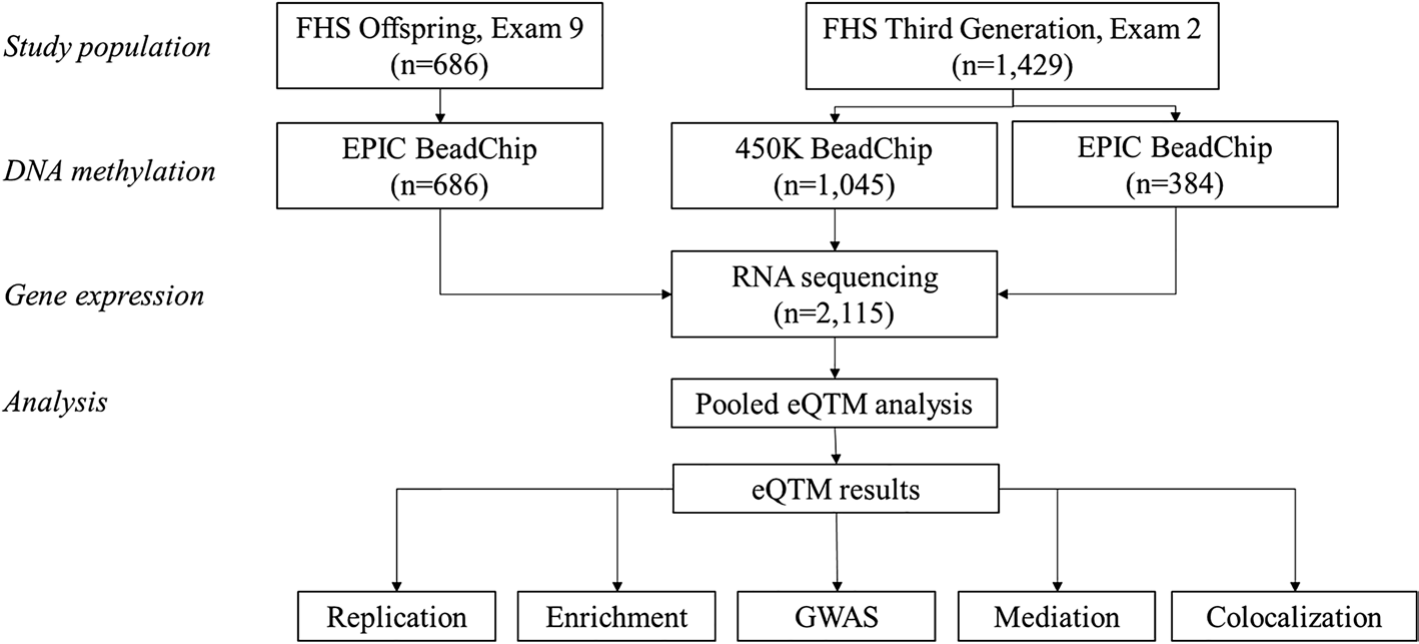
Overview of study design.

An overview of the study design is presented in Fig. 1. Of 2115 FHS participants (48% women, age 54 ± 15 years) included in the eQTM analysis, 686 were from the FHS Offspring cohort and 1,429 were from the FHS Third Generation cohort (Table 1). The average BMI was 28.0 ± 5.6 kg/m^2^, while the average serum triglycerides concentration was 114 ± 78 mg/dL. Average fasting blood glucose concentration was 100 ± 21 mg/dL.

**Table 1:** Characteristics of FHS participants.

	Total sample (n = 2115)	Offspring (n = 686)	Third generation (n = 1429)
Age, years	54 ± 15	66 ± 8	45 ± 8
Sex, n(%) female	1,010 (48%)	323 (47%)	687 (48%)
BMI, kg/m2	28.0 ± 5.6	28.4 ± 5.6	27.8 ± 5.6
Waist circumference, inches	38.6 ± 5.9	39.7 ± 5.8	38.0 ± 5.9
Current smoking, n (%)	151 (7%)	32 (5%)	119 (8%)
Alcohol intake, g/day	10 ± 15	7 ± 14	11 ± 15
Systolic blood pressure, mmHg	120 ± 16	128 ± 16	116 ± 14
Diastolic blood pressure, mmHg	74 ± 10	74 ± 10	74 ± 10
Fasting blood glucose, mg/dL [mmol/L]	100 ± 21 [5.53 ± 1.17]	106 ± 20 [5.9 ± 1.1]	97 ± 22 [5.4 ± 1.2]
Diabetes, n (%)	86 (4%)	41 (6%)	45 (3%)
LDL cholesterol, mg/dL	102 ± 31	107 ± 31	104 ± 30
HDL cholesterol, mg/dL	60 ± 18	58 ± 19	60 ± 17
Total cholesterol, mg/dL	185 ± 36	188 ± 36	186 ± 35
Serum triglycerides, mg/dL	114 ± 78	119 ± 70	113 ± 83
White blood cell count, predicted	6.0 ± 1.6	6.1 ± 1.2	6.0 ± 1.5
Platelet count, predicted	241 ± 56	252 ± 36	246.8 ± 51.1
Neutrophil percent predicted	59.5 ± 7.8	59.3 ± 7.3	58.7 ± 7.5
Lymphocyte percent predicted	27.8 ± 7.1	27.6 ± 7.0	28.8 ± 6.7
Monocyte percent predicted	8.8 ± 2.1	9.2 ± 1.8	8.7 ± 2.0
Eosinophils percent predicted	3.1 ± 1.8	3.1 ± 1.6	3.0 ± 1.7
Basophils percent predicted	0.75 ± 0.37	0.78 ± 0.21	0.81 ± 0.36

### Gene-level eQTM results

We defined *cis* CpG-transcript pairs as those where the CpG site and transcription start site were within 1 Mb of one another. We identified 70,047 significant *cis* CpG-transcript pairs (33,385 unique CpGs; 8534 unique eGenes) at *p* ≤ 1E−7; the top 10,000 most significant *cis* CpG transcript pairs are presented in Supplemental Table 1. The majority (49,280; 70%) of the significant *cis* CpG-transcript pairs involved transcripts of protein-coding genes, while seven percent (4971) were annotated to lncRNAs. The 8534 unique eGenes corresponded to 6693 unique lead CpGs, i.e. the CpG site that is most significantly associated with that eGene in an eQTM CpG-transcript pair. DNA methylation of the lead CpG site was associated with increased *cis* expression of 2909 (34%) eGenes, and with decreased *cis* expression of 5625 (66%) eGenes.

We additionally defined *trans* CpG-transcript pairs as those where the CpG site and transcription start site were > 1 Mb from one another, and we identified 246,667 significant *trans* CpG-transcript pairs (12,637 unique CpGs, 4300 unique eGenes) at *p* ≤ 1E−14; the top 10,000 most significant *trans* CpG-transcript pairs are presented in Supplemental Table 2. The majority (210,944; 86%) of the significant *trans* CpG-transcript pairs involved transcripts of protein-coding genes, and 11,271 (5%) involved expression of lncRNAs. The 4300 unique eGenes identified corresponded with 1139 lead CpGs. DNA methylation of the lead CpG site was associated with increased *trans* expression of 1566 (36%) eGenes, and with decreased *trans* expression of 2734 (64%) eGenes.

A QQ plot comparing observed to expected *p*-values across all *cis* and *trans* CpG-transcript pairs is presented in Supplemental Fig. 1, showing an abundance of significant results and no genomic inflation (inflation λ = 0.998).

### Gene-level eQTM internal validation

To conduct internal validation, we divided our total sample into two equal-sized subsets: the first included all participants who had DNA methylation assayed by the 450 K platform (discovery *n* = 1045), and the second included participants who had DNA methylation assayed by the EPIC platform (validation *n* = 1070). This internal validation analysis identified 40,148 significant *cis* CpG-transcript pairs in the discovery 450 K samples at *p* ≤ 1E−7. Of these, 79% (31,840 CpG-transcript pairs) were significant in the validation EPIC platform at a Bonferroni-corrected *p*-value ≤ 0.05/40,148. We also identified 241,323 *trans* CpG-transcript pairs in the discovery sample at *p* ≤ 1E−14, and of these, 31% (74,405 CpG-transcript pairs) were significant in the validation EPIC platform at a Bonferroni-corrected *p*-value ≤ 0.05/241,323.

### Replication of CpG-transcript pairs

A recent eQTM analysis of nasal epithelial cells by Kim et al.^1^ identified 16,867 significant CpG-transcript pairs using the Illumina HumanMethylation450K methylation data and RNA-seq gene expression data. We mapped 8481 CpG sites and 3331 gene transcripts to those included in our eQTM analyses, which corresponded to 15,158 CpG-transcript pairs that we subsequently compared with our significant *cis* and *trans* eQTM results. Of these, 1192 of 2703 (44%) *cis* CpG-transcript pairs replicated in our study at ***p*** ≤ 1E−7 with matching effect directionality. In contrast, only 41 of 6,165 (0.67%) *trans* CpG-transcript pairs identified in Kim et al. replicated in our study at *p* ≤ 1E−14 with matching effect directionality.

For the most significant *cis* and *trans* CpG-transcript pairs, we identified the top 1000 most significant unique eGenes and their lead CpG. We evaluated these pairs for replication in two independent external cohorts: a cohort of 1248 participants with European ancestry (EA), African ancestry (AA), and Hispanic ancestry (HA) from the Women’s Health Initiative (WHI), and a cohort of 521 Black participants from the Jackson Heart Study (JHS). Replication results for *cis* CpG-transcript pairs in both cohorts are presented in Supplemental Table 3, while replication results for *trans* CpG-transcript pairs in both cohorts are presented in Supplemental Table 4.

In the WHI, all but one of the 1000 *cis* CpG-transcript pairs from discovery in FHS replicated at a nominally significant *p*-value of 0.05 with matching effect directionality, while 997 replicated at *p* < 1E−7 with matching effect directionality. Furthermore, 973 of 1000 *trans* CpG-transcript pairs from FHS discovery replicated at a nominally significant *p*-value of 0.05 with matching effect directionality, while 926 *trans* CpG-transcript pairs replicated at *p* < 1E−14 with matching effect directionality.

In the JHS, 697 of 820 (85%) *cis* CpG-transcript pairs from FHS discovery replicated at a nominally significant *p*-value of 0.05 with matching effect directionality, while 455 (55%) replicated at *p* < 1E−7 with matching effect directionality. Furthermore, 722 of 880 (82%) *trans* CpG-transcript pairs from FHS discovery replicated at a nominally significant *p*-value of 0.05 with matching effect directionality, while 228 (26%) replicated at *p* < 1E−14 with matching effect directionality.

### Gene ontology

We evaluated gene ontology terms to identify specific biological and cellular pathways that were enriched among the top 1000 unique *cis* and *trans* eGenes^13,14^. The top 1000 unique *cis* eGenes were enriched in 33 pathways related to cell signaling and adhesion at a false discovery rate (FDR) ≤ 0.05 (Supplemental Table  5). The top 1000 unique *trans* eGenes were enriched in 582 pathways related to the activation of the immune response (Supplemental Table 6).

### GWAS enrichment

We examined the overlap of the significant eQTM CpG-transcript pairs with gene-trait associations in the National Human Genome Research Institute (NHGRI)-European Bioinformatics Institute (EBI) GWAS catalog^15^. Using lead eQTL genetic variants for eQTM transcripts in enrichment analysis, the *cis* eGenes were enriched for genes associated with 1208 traits in the GWAS catalog at an enrichment FDR ≤ 0.05 (Supplemental Table  7). The *trans* eGenes were enriched for genes associated with 1191 traits in the GWAS catalog at an enrichment FDR ≤ 0.05 (Supplemental Table 8).

### Genomic feature enrichment

We evaluated enrichment of the CpG sites included in significant CpG-transcript pairs for 17 specific genomic features, including CpG islands, shores, bodies, and differentially methylated regions (DMRs). Significant *cis* CpG-transcript pairs were significantly enriched for 15 of 17 genomic features at an enrichment FDR ≤ 0.05 (Supplemental Table 9), with the top most significant features being upstream CpG shores, CpG islands, downstream CpG shores, DNase hypersensitivity sites (DHS), and gene bodies. Significant *trans* CpG-transcript pairs were significantly enriched for 13 of 17 genomic features at an enrichment FDR ≤ 0.05 (Supplemental Table 10), with the top most significant features being CpG islands, DHS, gene bodies, regions within 200 bases upstream of the transcription start site (TSS), and enhancers.

### eQTM CpG-transcript associations

To illustrate the association between DNA methylation at specific CpG sites and gene expression, we identified the top five most significant *cis* CpG-transcript pairs and the top five most significant *trans* CpG-transcript pairs and plotted the associations for each pair (Supplemental Figs. 2 through 11). In general, these figures show a negative association between DNA methylation and gene expression; the only CpG-transcript pair to show a positive association is the *trans* relationship between methylation at cg13704117 (*KIAA1267)* and expression of ENSG00000214425.7 (*LRRC37A4P)* (Supplemental Fig. 11). The CpG-transcript associations in Supplemental Figs. 6, 6, and 8 exhibit bimodal distributions that were not attributable to any nearby SNPs (i.e., within 100 bp) in conditional analysis. rs115687047, located near cg04071440, did not contribute to the bimodality of its *cis* association with ENSG00000204644.9 (*ZFP57*) shown in Supplemental Fig. 4 (*p* = 0.15). rs72989301, located near cg17901463 (*GSTM1*), was significantly associated with expression of ENSG00000134184.12 *(GSTM1)* (*p* = 1.2E−26), but a significant *cis* association between the CpG and transcript remained (*p* < 1E−308) as shown in Supplemental Fig. 6. Finally, the SNPs rs1619178 and rs3113782 are located near cg20262684 and have a linkage disequlibrium of 1; they did not contribute to the bimodality of its *trans* association with ENSG00000185304.15 (*RGPD2*) shown in Supplemental Fig. 8 (*p* = 0.75).

**Figure 2:**
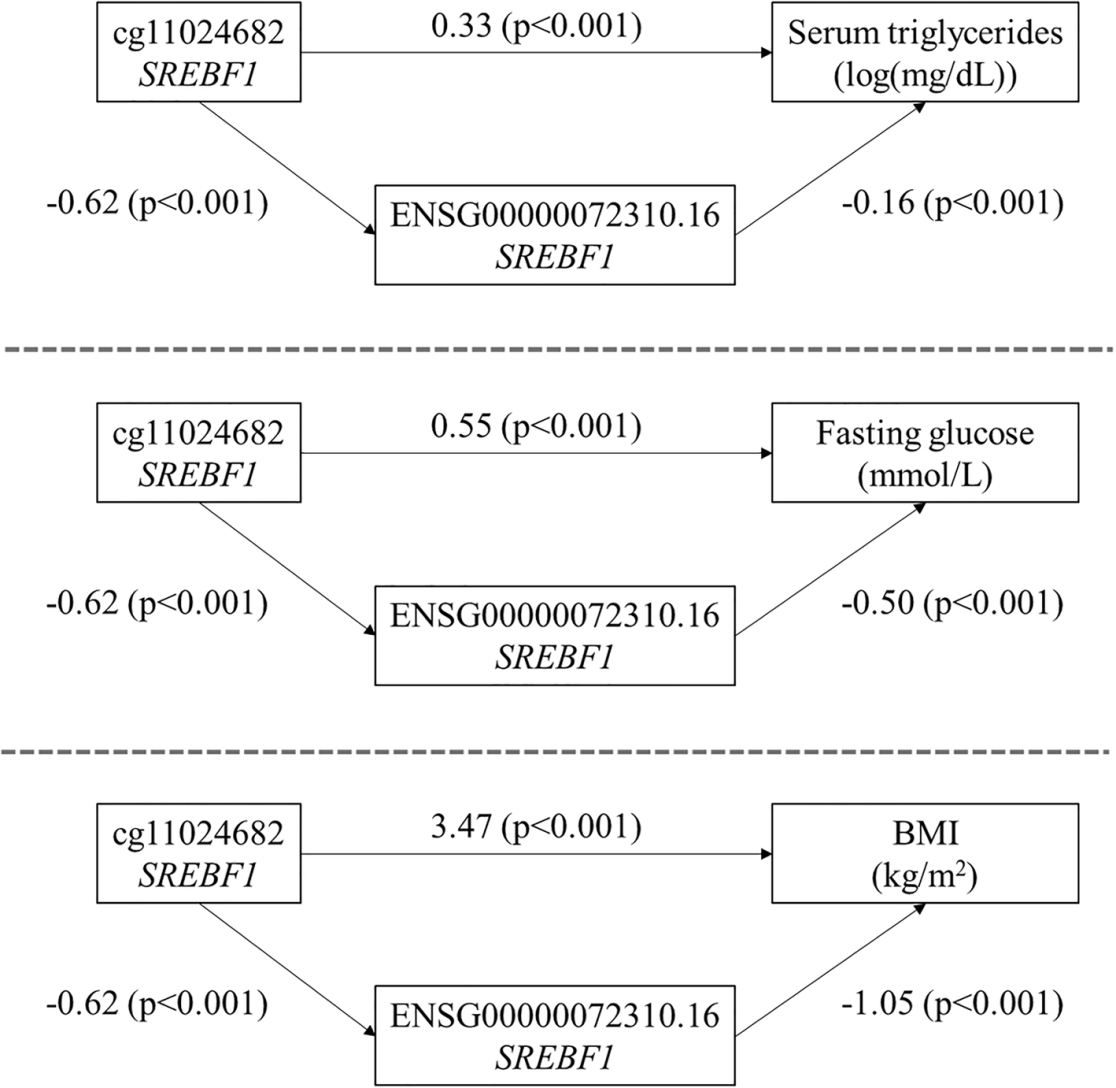
Pathways showing *cis* mediated effects of DNA methylation at cg11024682 on serum triglycerides, fasting blood glucose, and BMI through the expression of *SREBF1*. The effects of DNA methylation at the CpG site cg11024682 on all three cardiometabolic traits of interest are mediated through *cis* effects on *SREBF1* expression. Coefficients presented in the figure represent the change in gene expression associated with a 10% increase in DNA methylation; the change in serum triglycerides (log(mg/dL)), fasting blood glucose (mmol/L), or BMI (kg/m^2^) associated with a 10% increase in DNA methylation; or the change in serum triglycerides (log(mg/dL)), fasting blood glucose (mmol/L), or BMI (kg/m^2^) associated with a 1-unit increase in gene expression.

### eQTM CpG-transcript pairs in mediation analysis of cardiometabolic traits

To illustrate the application of the eQTM resource we generated, we provide examples from the associations of significant *cis* CpG-transcript pairs with three cardiometabolic traits: serum triglycerides, fasting blood glucose, and body mass index (BMI). Specifically, we first evaluated the mediated effect of DNA methylation on the clinical phenotype through the expression of the linked eGene. To identify candidate CpG-transcript pairs to include in mediation analysis, we identified (1) the overlap of significant *cis* CpG sites with published CpG-trait associations from the MRC Integrative Epidemiology Unit (IEU) EWAS catalog (http://www.ewascatalog.org/)^16^, and (2) significant results from de novo transcriptome-wide association studies (TWAS) of RNA-seq gene expression data and log-transformed serum triglycerides, fasting blood glucose, and BMI in the FHS. The strategy of aligning trait-associated CpGs with corresponding trait-associated transcripts identified 14 CpG-transcript pairs associated with serum triglycerides, 36 associated with fasting glucose, and 153 associated with BMI. These pairs and their corresponding traits were then tested for the following mediation effects: (1) the total effect of DNA methylation on the cardiometabolic trait, (2) the direct effect of DNA methylation on the cardiometabolic trait independent of gene transcript expression, and (3) the mediated effect of DNA methylation on the cardiometabolic trait through gene transcript expression. Results are summarized in Supplemental Table 11. As an example, Fig. 2 illustrates significant mediation of the effects of DNA methylation at the CpG site cg11024682 on serum triglycerides, fasting blood glucose, and BMI via expression of the *SREBF1* gene. The proportion of the association between DNA methylation and the three cardiometabolic traits mediated by *SREBF1* expression ranged from 0.16 to 0.36.

Gene transcript expression mediated the effect of DNA methylation on serum triglycerides (log-transformed) for ten of the 14 CpG-transcript pairs. Significant mediation of DNA methylation on gene expression was observed for the expression of *RNASET2, SREBF1, ABCG1,* and *TOM1L2* at *p* ≤ 0.05.

Gene transcript expression significantly mediated the effect of DNA methylation on fasting blood glucose for 24 of the 36 CpG-transcript pairs, of which six had direct and mediated effects in opposite directions (e.g., a negative direct effect and a positive mediated effect). The greatest proportions of the CpG-fasting glucose associations mediated by gene expression (*p*  < 2E−16 for all) were observed for the expression of *KLRF1* (proportion mediated = 0.87), *TRAPPC2B* (proportion mediated = 0.82), *NKIRAS2* (proportion mediated = 0.74), and *RRP12* (proportion mediated = 0.52).

Finally, gene transcript expression significantly mediated the effect of DNA methylation on BMI for 117 of the 153 CpG-transcript pairs, of which 55 had direct and mediated effects in opposite directions. For the CpG-transcript pairs where the direct and mediated effects were in the same direction, the greatest proportions of the CpG-BMI associations mediated by gene expression (*p* < 2E−16 for all) were observed for the expression of *TSPYL1* (proportion mediated = 0.98), *PLD3* (proportion mediated = 0.94), and *FAS* (proportion mediated = 0.80). Additionally, two CpG-transcript pairs showed significant mediation of the CpG-BMI association through expression of lncRNA genes *BX284668.5* and *LINC00996*.

### eQTM CpG-transcript pairs in colocalization analysis of cardiometabolic traits

**Figure 3:**
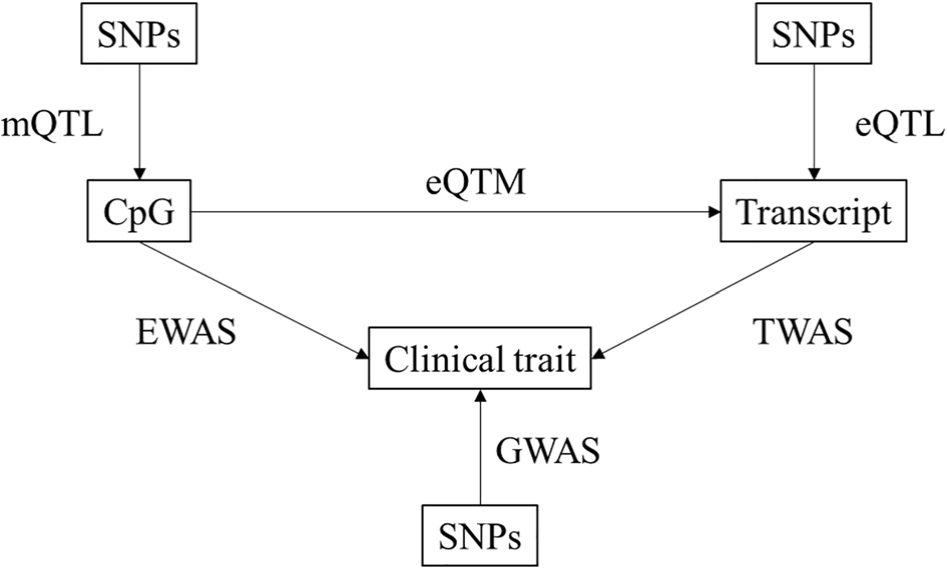
Overview of approach to combining eQTM results with other data sources for clinical and translational applications.

We conducted a Bayesian colocalization analysis to evaluate whether the *cis*-CpG-transcript eQTM pairs associated with serum log triglycerides, fasting blood glucose, and BMI were regulated by shared genetic variants. The MRC IEU EWAS catalog was used to identify CpG sites associated with each of the clinical phenotypes of interest, and three sources of data were used for colocalization analysis (Fig. 3). Among significant serum triglycerides-associated CpG sites identified in the MRC IEU EWAS catalog, 15 overlapped with CpGs from significant *cis* CpG-transcript pairs where the CpG and gene transcript also shared at least one SNP (i.e., the eQTL variant for the transcript matched the mQTL variant for its paired CpG). Colocalization results for the 15 *cis* eQTM CpG-transcript pairs, which included 12 unique CpG sites associated with 4876 significant *cis-*mQTL variants and 11 unique gene transcripts associated with 6,327 significant *cis*-eQTL variants, are presented in Supplemental Table 12. The probability of colocalization H4 ≥ 80% was observed between the CpG and transcript SNPs for ten eQTM CpG-transcript pairs. The SNP rs7215055 was associated with serum triglycerides^17^ and was also associated with methylation of CpG cg11024682 and expression of gene *ATPAF2* (probability of colocalization H4 = 100%).

Among significant fasting glucose-associated CpG sites identified in the MRC IEU EWAS catalog, 138 overlapped with CpGs from significant *cis-*CpG-transcript pairs where the CpG and gene transcript also shared at least one SNP. Colocalization results for the 138 *cis-*eQTM CpG-transcript pairs, which included 109 unique CpG sites associated with 38,758 significant *cis-*mQTL variants and 172 unique gene transcripts associated with 100,546 significant *cis-*eQTL variants, are presented in Supplemental Table 13. Probability of colocalization ≥ 80% was observed between the CpG and transcript SNPs for 51 eQTM CpG-transcript pairs. There was no overlap of SNPs identified in a GWAS of fasting glucose^18^ with both mQTL and eQTL variants for CpG-transcript pairs, thus further colocalization analyses were not carried out for this trait.

Finally, among significant BMI-associated CpG sites identified in the MRC IEU EWAS catalog, 207 overlapped with CpGs from significant *cis* CpG-transcript pairs where the CpG and gene transcript also shared at least one SNP. Colocalization results for the 207 *cis-*eQTM CpG-transcript pairs, which included 134 unique CpG sites associated with 82,823 significant *cis-*mQTL variants and 182 unique gene transcripts associated with 232,511 significant *cis-*eQTL variants, are presented in Supplemental Table 14. Probability of colocalization ≥ 80% was observed between the CpG and transcript for 67 eQTM CpG-transcript pairs. The SNPs rs3817334, rs10838738, and rs1064608, which were associated with BMI^19^, were also mQTL genetic variants associated with the CpG cg17580616 and eQTL genetic variants associated with the gene *ACP2* (probability of colocalization H4 = 100%).

## Discussion

Our study of DNA methylation in relation to gene expression in 2115 FHS participants identified over 70,000 significant *cis* CpG-transcript pairs and over 246,000 significant *trans* CpG-transcript pairs. We generated a comprehensive database of whole blood *cis* and *trans* eQTM CpG-transcript pairs that can be used to investigate disease mechanisms, pathways, and processes. We previously evaluated eQTM CpG-transcript pairs in the FHS using the Illumina 450 K DNA methylation array in conjunction with array-based gene expression data^7^. The present study expands on this prior work by incorporating more extensive DNA methylation profiling (Illumina EPIC DNA methylation array) and RNA-seq expression, resulting in a powerful resource for capturing associations between DNA methylation and gene expression. Not only did we identify four times as many *cis*-eQTM CpG-transcript pairs (70,047 vs. 16,416) and 24% more *trans*-pairs (246,667 vs. 198,960) than we previously identified using array-based gene expression data^7^, but we also provide eQTM CpG-transcript pairs for the expression of lncRNAs.

DNA methylation can alter the regulation of gene transcription in multiple ways, such as aiding in the recruitment of proteins involved in increasing gene expression or inhibiting transcription factor binding to a specific DNA sequence. At promoter sites, DNA methylation generally precludes transcription directly by blocking the binding of transcriptional activators or indirectly by recruiting methyl-binding proteins and co-repressor complexes. In this study, we used eQTM analysis to identify numerous CpG sites that, when methylated, are associated with *cis* and *trans* eGene expression. Associations between SNPs and DNA methylation (i.e., mQTL associations) may contribute to bimodal distributions in eQTM CpG-transcript associations, many of which were not attributable to any nearby SNPs (i.e., there was no evidence of SNP-on-CpG effects^20^ contributing to bimodality). Indeed, conditional analyses of nearby SNPs on select associations showed that the CpG-transcript associations are still highly significant. Furthermore, to showcase the potential clinical utility of our findings, we evaluated the role of our eQTM resource when applied to the analysis of three cardiometabolic risk factors—serum triglycerides, fasting blood glucose, and BMI—in mediation and colocalization analyses. We illustrate specific examples of how the eQTM resource can be used to suggest molecular mechanisms linking DNA methylation to clinical phenotypes: Mediation analyses identified putative pathways by which DNA methylation is associated with clinical traits in EWAS, and colocalization analysis suggested shared genetic regulation of CpGs, transcripts, and clinical traits. For example, the results of our mediation analysis showed that expression of the genes *ABCG1* and *SREBF1* mediates the association between DNA methylation and all three clinical traits.

While the eQTM resource we created is robust and highly replicable, several limitations must be noted. First, FHS is an observational study consisting of participants of predominantly European ancestry; however, we provide evidence of substantial independent external replication of these results, including among individuals with African ancestry from the JHS. Second, while the use of DNA methylation and gene expression data derived from whole blood samples makes our methods more accessible and more easily replicated in other study cohorts, evaluation of CpG-transcript pairs in other tissues may be more relevant to specific disease processes. Additionally, these analyses do not account for linkage disequilibrium and other factors that may correlate proximal CpGs with one another. Finally, this study does not examine the effect of environmental factors on these associations; lifestyle factors such as cigarette smoking are known to affect DNA methylation and gene expression^7,21–23^. Future eQTM research may incorporate environmental data to better understand the epigenetic effects of environmental exposures.

## Conclusions

We created a powerful eQTM resource that leverage DNA methylation and RNA-seq data to characterize associations of DNA methylation with gene expression. We provide proof of concept that eQTM resources can be leveraged to explore molecular mechanisms of disease.

## Methods

### Cohort description

DNA methylation and RNA-seq data were collected from 2115 participants in the FHS Offspring (n = 686) and Third Generation (n = 1429) cohorts. Peripheral whole blood samples were collected in the fasting state at the ninth examination cycle from FHS Offspring participants (2011–2014)^24^ and at the second examination cycle (2006–2009)^25^ from FHS Third Generation participants. All study protocols were approved by the Boston Medical Center Institutional Review Board and performed in accordance with the Declaration of Helsinki and relevant regulations, and all study participants provided their written informed consent.

### DNA methylation data collection

Buffy coats were isolated from whole blood samples and prepared with bisulfite conversion for the DNA methylation assays. Samples from 1045 FHS Third Generation participants were assayed for DNA methylation using the Infinium HumanMethylation450 BeadChip (Illumina Inc., San Diego, CA). Samples from the remaining 384 FHS Third Generation participants and all FHS Offspring participants were assayed for DNA methylation using the Infinium MethylationEPIC BeadChip (Illumina Inc., San Diego, CA). Methylation probes on autosomal chromosomes were analyzed while probes containing polymorphic SNPs were excluded.

### DNA methylation data pre-processing

DNA methylation data were pooled across both the HumanMethylation450 and the MethylationEPIC platforms, which shared 452,568 CpG loci. In addition to the data captured at these loci, samples assayed by the HumanMethylation450 platform had methylation data for 32,945 additional CpG sites, while those assayed by the MethylationEPIC platform had methylation data for 413,524 additional CpG sites.

Quality control (QC) and normalization were performed on the DNA methylation β values using the “dasen” function in the R *wateRmelon* package (version 1.16.0)^26^. Normalized β values were then residualized after accounting for batch effects, row effects, column effects, and four PCs constructed from the normalized β values. These residualized β values were subsequently winsorized at the mean ± 3 * standard deviation (SD) of the distribution for each probe.

Ten principal components (PCs) representing technical confounders were identified from the winsorized residuals. These ten PCs and the winsorized β values representing the proportion of methylation per CpG locus were subsequently used in statistical analyses.

### RNA-seq data collection

Whole blood samples were collected in PAXgene tubes (PreAnalytiX, Hombrechtikon, Switzerland). Isolation of total RNA was performed according to standard protocols (Asuragen, Inc., Austin, TX) using the PAXgene Blood RNA System Kit as described previously^27,28^ RNA-seq was performed in accordance with TOPMed protocols (University of Washington Northwest Genomics Center), and the mRNA-seq library was generated using the Illumina TruSeq (Illumina Inc., San Diego, CA). Stranded mRNA kit and sequencing was performed using the Illumina NovaSeq system (Illumina Inc., San Diego, CA). Gene expression was evaluated using RNA SeQC version 2.3.3^29^ and transcript expression was evaluated using RSEM version 1.3.1^30^.

### RNA-seq data pre-processing

Gene expression data were normalized using the trimmed mean of M-values (TMM) approach in the R *edgeR* package^31^. A log2 transformation was applied to the TMM-normalized values after addition of 1 unit to avoid taking log of zero. This normalized gene expression value was residualized to account for batch effects, RNA concentration, and RNA integrity number.

### Statistical methods: eQTM analysis

We identified PCs from the residualized DNA methylation and gene expression data and performed an extensive search of the number of PCs to optimize the cross-validated replication of the CpG-transcript pairs. This study identified ten PCs for the DNA methylation data (percent of variation 53.7%) and five PCs for the gene expression data (percent of variation 51.6%).

We then calculated the association between gene-level CpG sites and gene transcripts to identify significant eQTM CpG-transcript pairs. For each CpG-transcript pair, residualized gene expression was modeled as the outcome with residualized DNA methylation β values as the primary explanatory variable. These models were adjusted for age, sex, white blood cell count, blood cell fraction^32^, platelet count, five gene expression PCs, and ten DNA methylation PCs. Each CpG-transcript pair association was calculated individually according to this model, which was repeated for each pair using Graphical Processing Units (GPUs) to accelerate computation. *Cis* gene-level CpG-transcript pairs were defined as those where the CpG site and the eGene (i.e., the gene transcript associated with the CpG) were within 1 Mb kb of one another and were deemed statistically significant at a Bonferroni-corrected *p*-value of 1E−7^33^. *Trans* pairs were defined as those where the CpG site and the eGene were more than 1 Mb apart. While an estimated Bonferroni *p*-value cutoff of 1E−7/50,000 gene transcripts was calculated, we deemed *trans* pairs as statistically significant at a stricter *p*-value of 1E−14 to reduce noise and false positives based on an examination of the genomic positions of CpG sites and gene transcripts. This *p*-value threshold optimized the tradeoff between the replication rate and the number of significant pairs. Results were annotated to the Illumina CpG reference database^34^ and to GENCODE 30.

### Replication of CpG-transcript pairs

In this study, we evaluated replication of the CpG-transcript eQTM pairs identified in two ways. First, we evaluated the overlap of significant *cis* and *trans* CpG-transcript pairs with a recent eQTM study that identified 16,867 significant CpG-transcript pairs in nasal epithelial cells (FDR < 0.01). Second, we identified the top 1000 most significant lead CpG sites and their associated eGenes and tested those eQTM pairs for statistical significance in two external and independent study cohorts: the Women’s Health Initiative (WHI) and the Jackson Heart Study (JHS). Both study cohorts completed analyses using DNA methylation data from the Infinium MethylationEPIC Beadchip (Illumina Inc., San Diego, CA) array processed in TOPMed. Background correction and normalization was done using the normal-exponential out-of-band (NOOB) method in the R *minfi* package^35,36^. Additionally, both study cohorts used estimated count gene expression data from RNA-SeQC^29^ from the TOPMed RNA-seq processing pipeline.

### Replication cohort: WHI

The WHI is a large prospective study of 161,808 postmenopausal women who enrolled into one of two study components between 1993 and 1998: the clinical trial of hormone therapy, calcium/vitamin D supplementation, and/or dietary modification, and the observational study. The present study included DNA methylation and RNA-seq data from 1248 WHI participants (818 with European ancestry, 338 with African ancestry, and 92 with Hispanic ancestry) who completed the Long Life Study follow-up examination between March 2012 and May 2013. All participants provided their written informed consent. Data cleaning was completed by self-reported race and ethnicity and the eQTM models were adjusted for age at sample collection, race/ethnicity, directly measured CBC counts, 10 expression PCs, and 15 methylation PCs.

### Replication cohort: JHS

The JHS is a population-based cohort study of cardiovascular risk factors and disease in 5306 African Americans. The present replication study included DNA methylation and RNA-seq data from 521 JHS participants. All participants provided written informed consent to use of genetic data.

Whole blood samples from the JHS baseline examination were assayed for DNA methylation using the EPIC array as previously described^37^. The COMBat method^38^ was used to correct the normalized DNA methylation β values for batch effects.

RNA-seq data from peripheral blood mononuclear cells (PBMCs; average read depth of 50M, University of Washington Northwest Genomics Center (NWGC)) were selected based on expression thresholds of 0.1 transcripts per million (TPM) and 6 expected read counts from RSEM version 1.3.1^30^ in at least 20% of samples. Gene expression values were inverse normal transformed across samples and probabilistic estimation of expression residuals (PEER) was used to generate 10 factors. Gene expression data was subsequently adjusted using age, sex, the top 10 JHS genotype PCs, and the 10 PEER factors.

After QC, 820 of the top 1000 *cis* CpG-transcript pairs evaluated for replication were available in this cohort; similarly, 880 of the top 1000 *trans* CpG-transcript pairs evaluated for replication were available in this cohort. eQTM analysis was completed for these pairs that passed QC after adjustment for the fixed effects of age, sex, and CBC, and for family ID as a random effect.

### Gene ontology

Entrez gene IDs for the eGenes were included in gene ontology analyses, and we used the function “goana” in the R package *limma*^39^ to identify biological, cellular, and molecular pathways enriched in the eGenes. Statistically significant pathways were identified at a false FDR ≤ 0.05.

### GWAS enrichment of clinical phenotypes associated with CpG-transcript pairs

The GWAS catalog included associations of 243,587 unique SNPs with 2960 unique clinical traits at *p* ≤ 5E−8; SNPs were mapped to the gene(s) in which they were located. We compiled a list of unique gene transcripts that were part of statistically significant CpG-transcript pairs, as well as the associations of those genes with clinical phenotypes in the GWAS catalog. We subsequently used a one-sided Fisher’s exact test to evaluate enrichment for each gene with each of the 2960 GWAS traits.

### Applications of eQTM resource: mediation analysis

Mediation analysis was performed using the function “mediate” in the R package *mediation* (version 4.5.0)^40^ to obtain total, direct, and mediated effects. This analysis provided the proportion of the total effect of the DNA methylation on the clinical phenotype mediated through gene expression. Results were divided by a factor of ten to obtain the effects associated with a 10% increase in DNA methylation of a CpG.

RNA-seq gene expression data for TWAS were residualized after adjustment for technical covariates and pedigree. All TWAS were then adjusted for age, sex, smoking status, alcohol use, white blood cell count, and predicted blood cell fraction^32^. The TWAS of serum log triglycerides was additionally adjusted for BMI and use of lipid medications, and the TWAS of fasting glucose was additionally adjusted for BMI and diabetes status. Diabetes status was defined as a fasting glucose concentration ≥ 126 mg/dL or use of diabetes medications.

### Applications of eQTM resource: colocalization analysis

For the CpG-transcript pairs evaluated in colocalization analysis, we used FHS *cis* mQTL and *cis* expression quantitative trait loci (eQTL) results to identify overlapping single nucleotide polymorphisms (SNPs) associated with the CpG and gene transcript, respectively. We then evaluated the overlap of *cis*-SNPs associated with CpG sites (mQTL) and *cis-*SNPs associated with gene transcripts (eQTL) with SNPs associated with serum log triglycerides^17^, fasting blood glucose^18^, and BMI^19^ in published genome-wide association studies (GWAS). We used the R package *coloc* (version 5.1.0)^41^ to quantify the probabilities (probability of colocalization H4) that a single genetic variant was associated with both DNA methylation and gene expression (i.e., colocalization of the mQTL and eQTL SNPs) and that a single genetic variant was associated with both DNA methylation and the clinical trait of interest (i.e., colocalization of the mQTL and GWAS SNPs).

### Supplementary Information

Supplementary Tables.

Supplementary Figures.

## Data Availability

The FHS DNA methylation and RNA-seq datasets analyzed in the current study are available at the database of Genotypes and Phenotypes (dbGaP) repository phs000007.v32.p13 (https://www.ncbi.nlm.nih.gov/projects/gap/cgi-bin/study.cgi?study_id=phs000007.v30.p11). JHS data are available on dbGaP at phs000286 (phenotype data) and phs000964 (TOPMed data). WHI data are available on dbGaP at phs001237.
