# Supplementary material for: Expression quantitative trait methylation analysis elucidates gene regulatory effects of DNA methylation: the Framingham Heart Study
Source: Sci Rep. 2023 Aug 10;13:12952. doi: 10.1038/s41598-023-39936-3 (PMC10415314; doi:10.1038/s41598-023-39936-3)

## Expression quantitative trait methylation analysis elucidates gene regulatory effects of DNA methylation: The Framingham Heart Study

Amena Keshawarz, Helena Bui, Roby Joehanes, Jiantao Ma, Chunyu Liu, Tianxiao Huan, Shih-Jen Hwang, Brandon Tejada, Meera Sooda, Paul Courchesne, Peter J. Munson, Cumhur Y. Demirkale, Chen Yao, Nancy L. Heard-Costa, Achilleas N. Pitsillides, Honghuan Lin, Ching-Ti Liu, Yuxuan Wang, Gina M. Peloso, Jessica Lundin, Jeffrey Haessler, Zhaohui Du, Michael Cho, Craig P. Hersh, Peter Castaldi, Laura M. Raffield, Jia Wen, Yun Li, Alexander P. Reiner, Mike Feolo, Nataliya Sharopova, Ramachandran S. Vasan, Dawn L. DeMeo, April P. Carson, Charles Kooperberg, Daniel Levy

Supplemental Figure 1. QQ plot showing relationship between observed and expected log_10_(p) values.


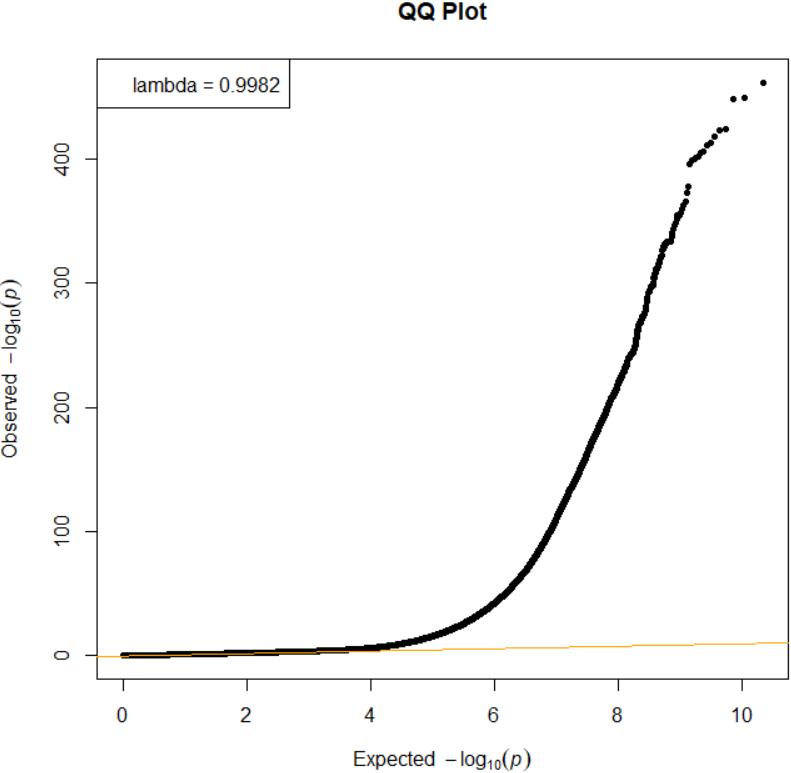


Supplemental Figure 2. *Cis* association of DNA methylation at cg21550016 and expression of ENSG00000189223.14.


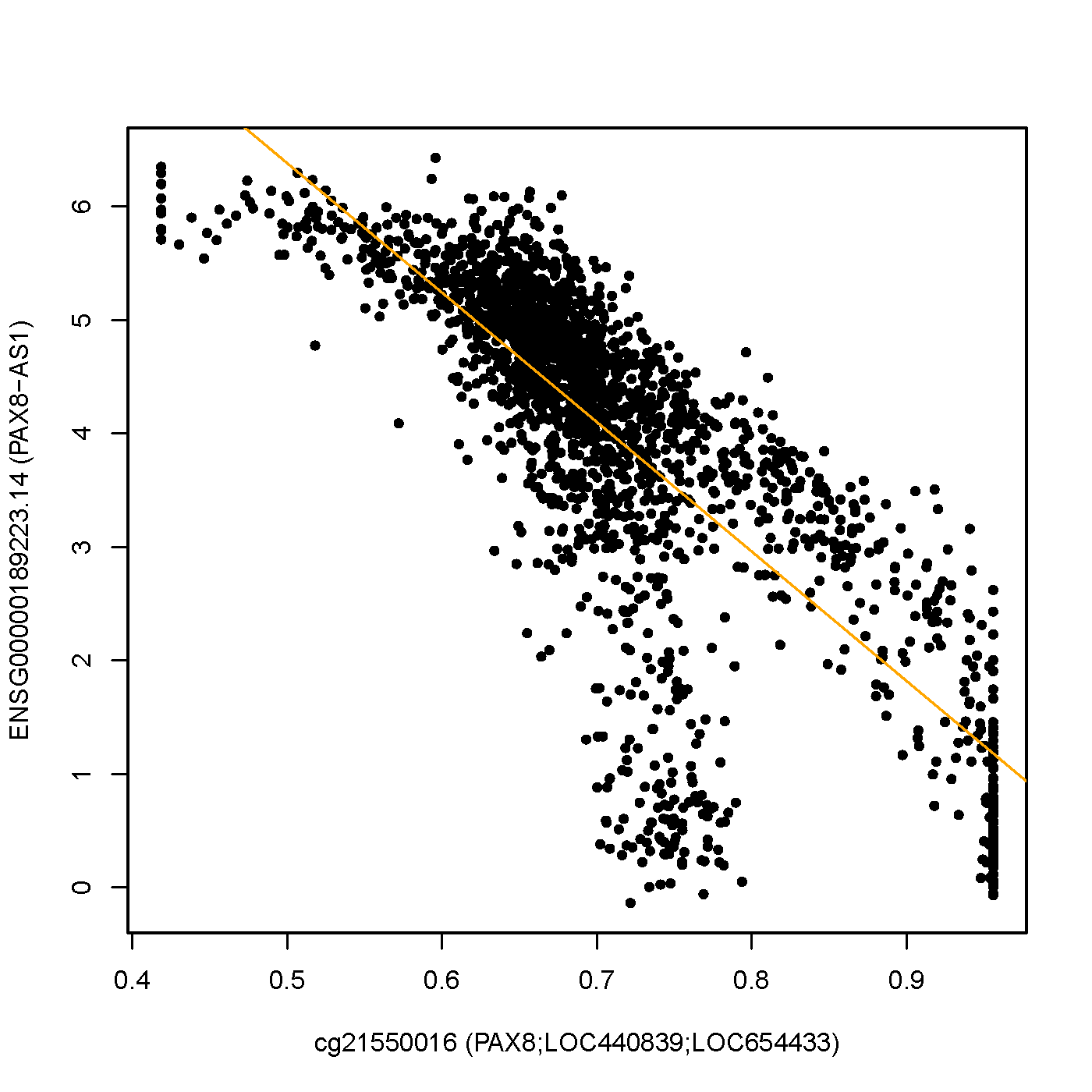


Supplemental Figure 3. *Cis* association of DNA methylation at cg07280731 and expression of ENSG00000007038.11.


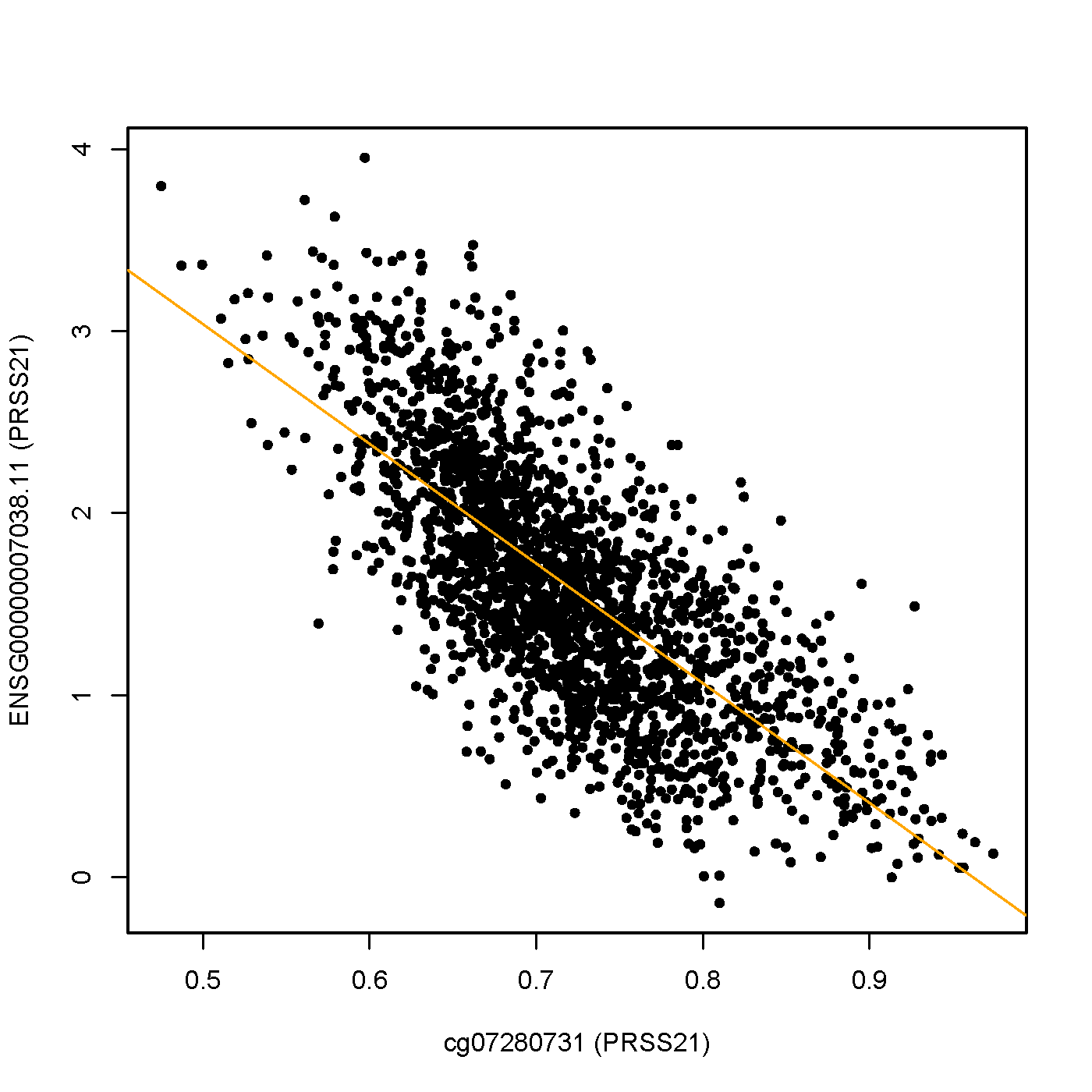


Supplemental Figure 4. *Cis* association of DNA methylation at cg04071440 and expression of ENSG00000204644.9.


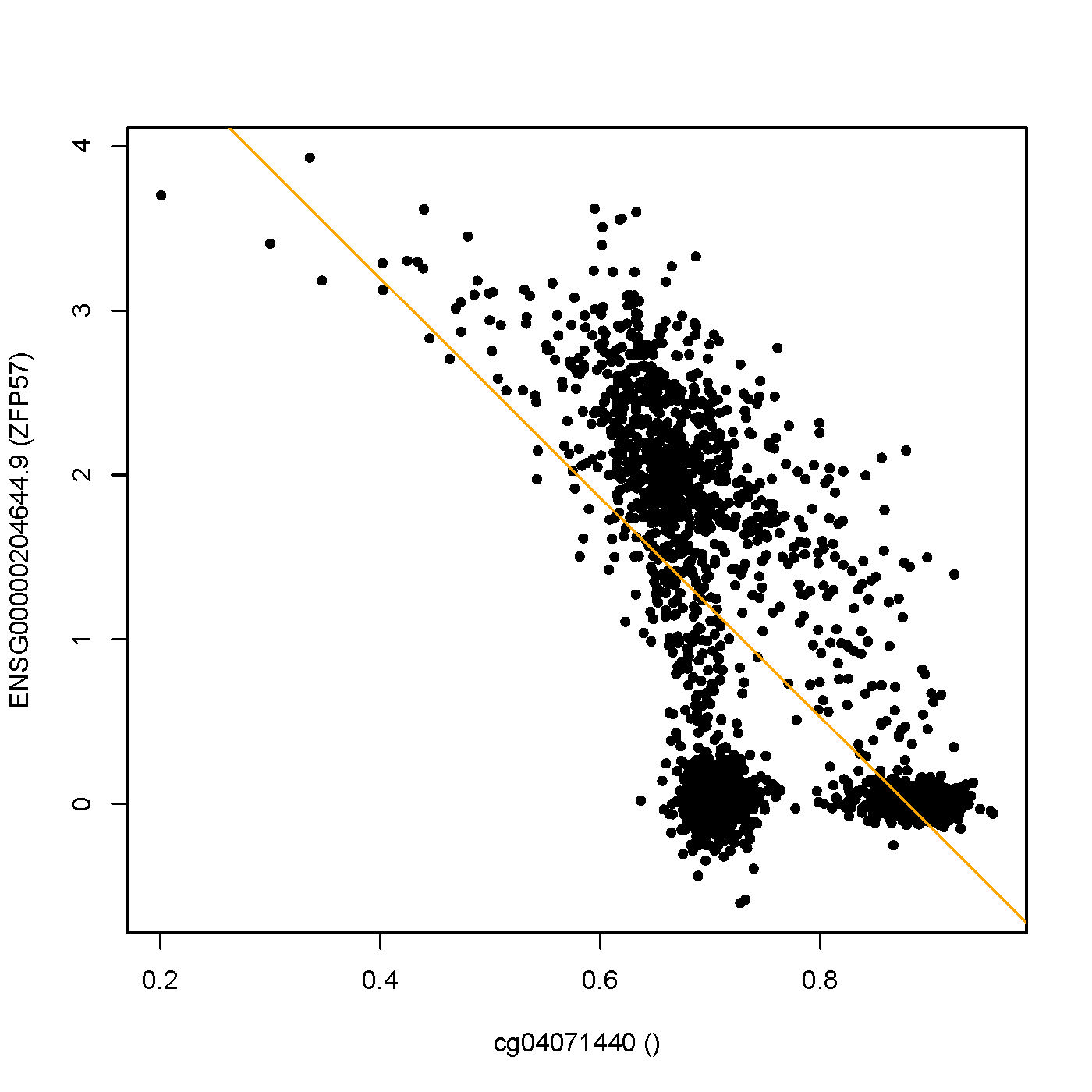


Supplemental Figure 5. *Cis* association of DNA methylation at cg11763394 and expression of ENSG00000189223.14.


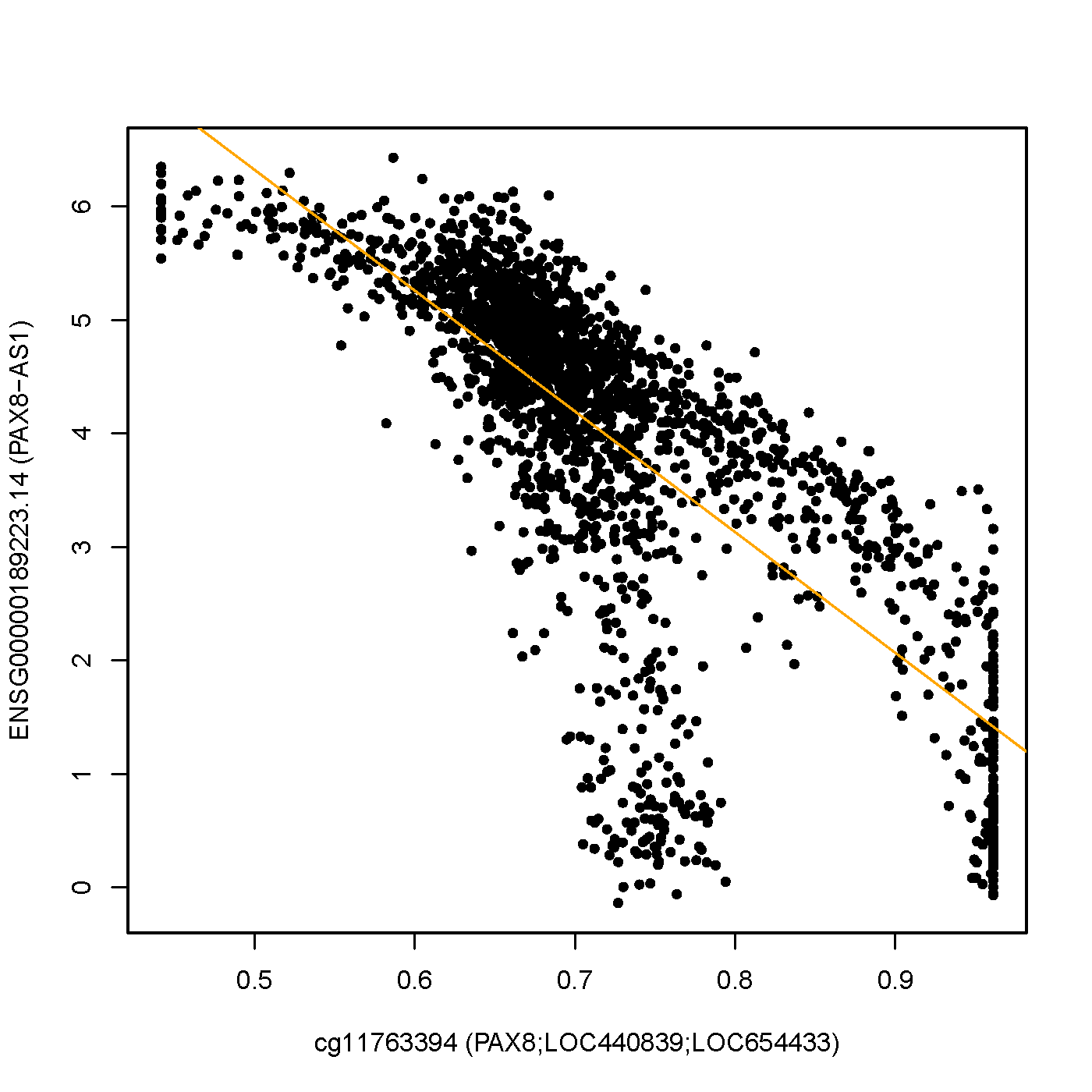


Supplemental Figure 6. *Cis* association of DNA methylation at cg17901463 and expression of ENSG00000134184.12.


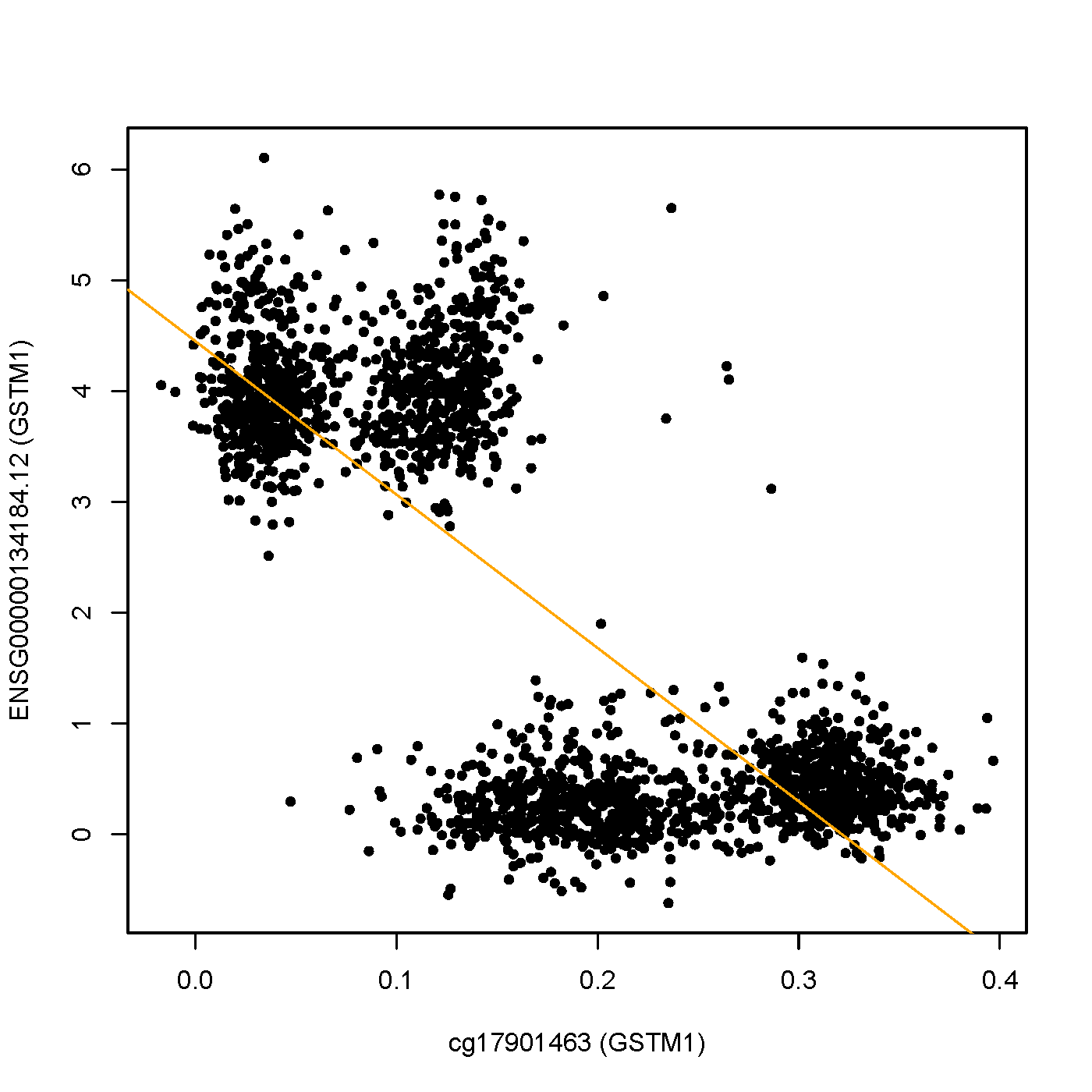


Supplemental Figure 7. *Trans* association between DNA methylation at cg15765885 and expression of ENSG00000185304.15.


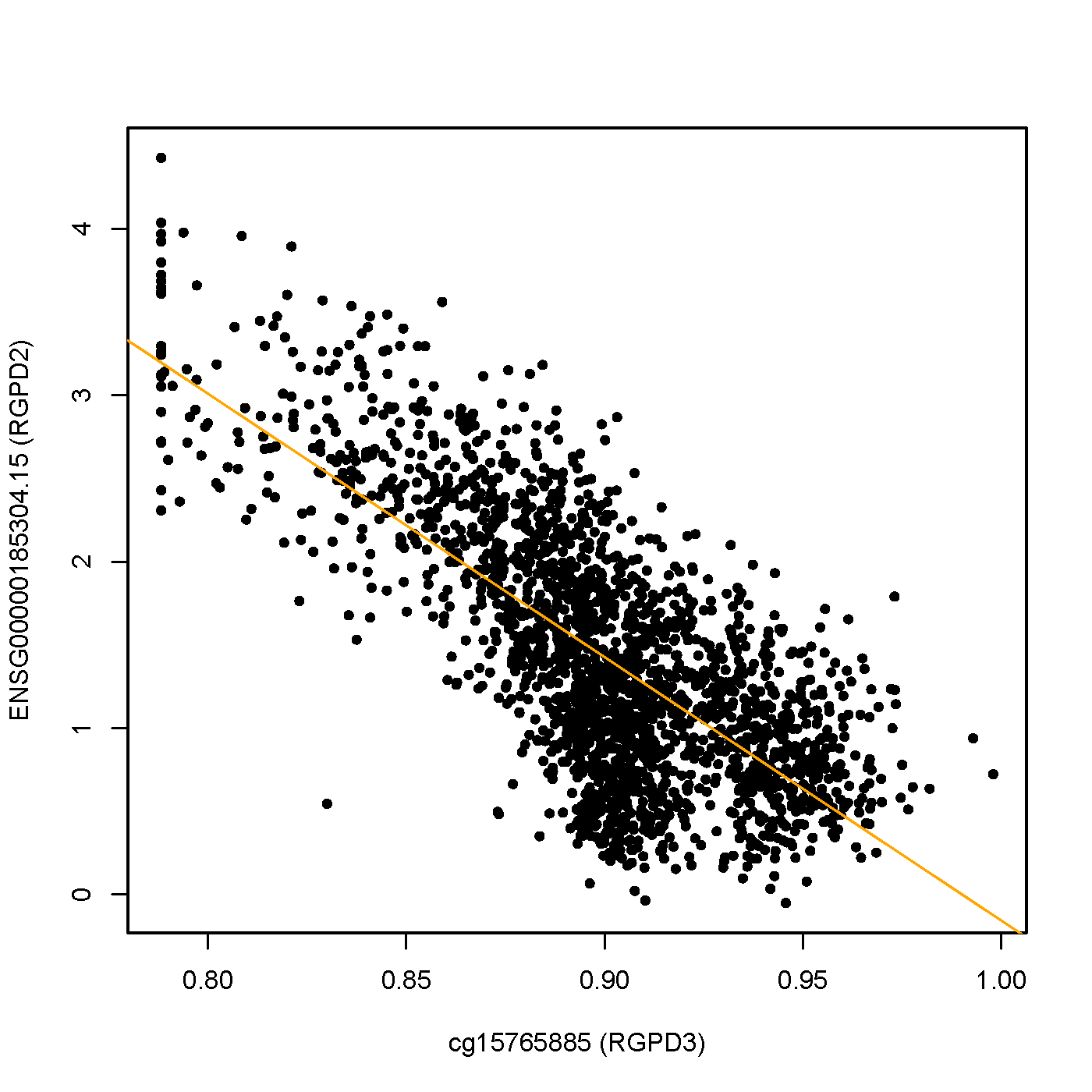


Supplemental Figure 8. *Trans* association between DNA methylation at cg20262684 and expression of ENSG00000185304.15.


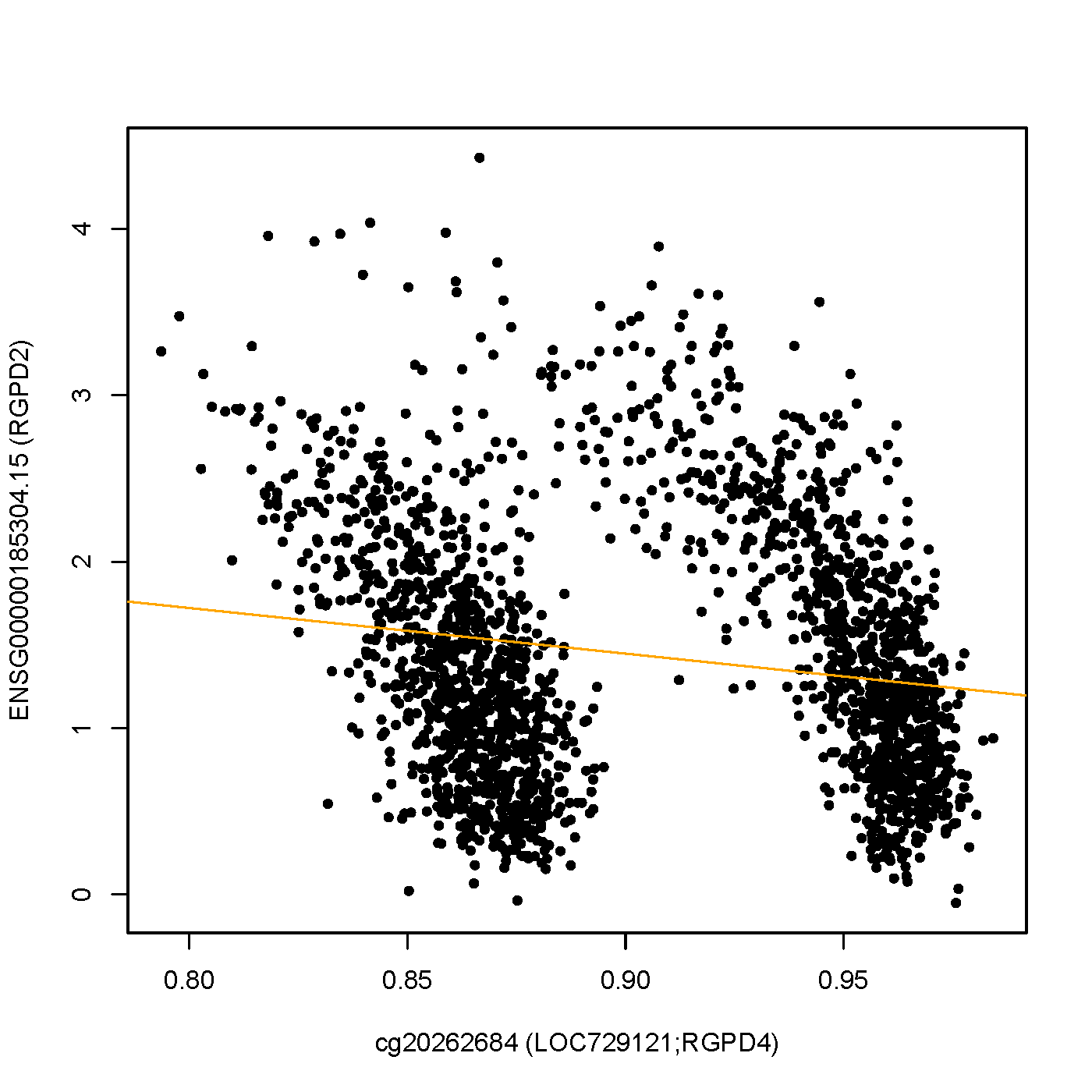


Supplemental Figure 9. *Trans* association between DNA methylation at cg17055585 and expression of ENSG00000257599.2.


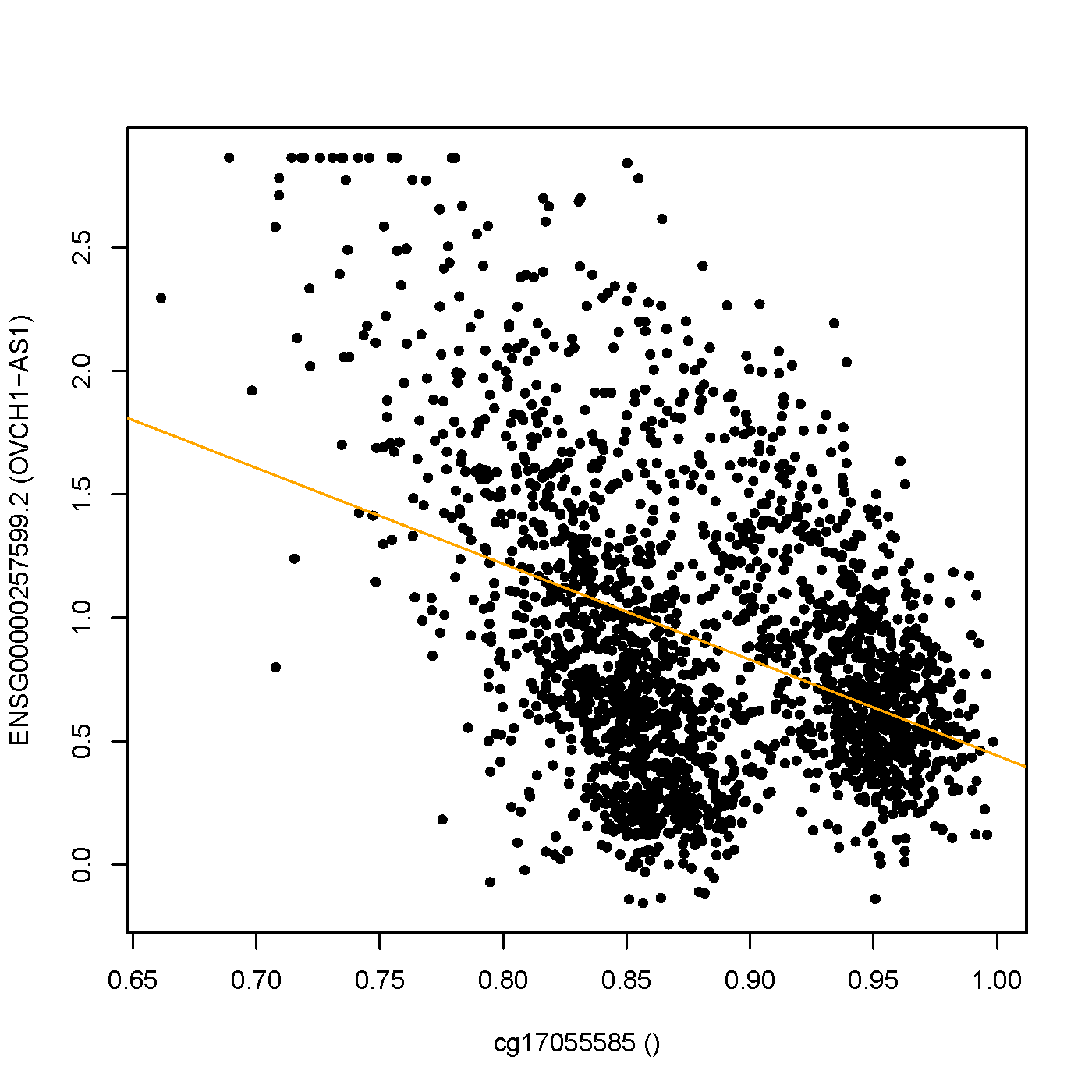


Supplemental Figure 10. *Trans* association between DNA methylation at cg05575921 and expression of ENSG00000154165.5.


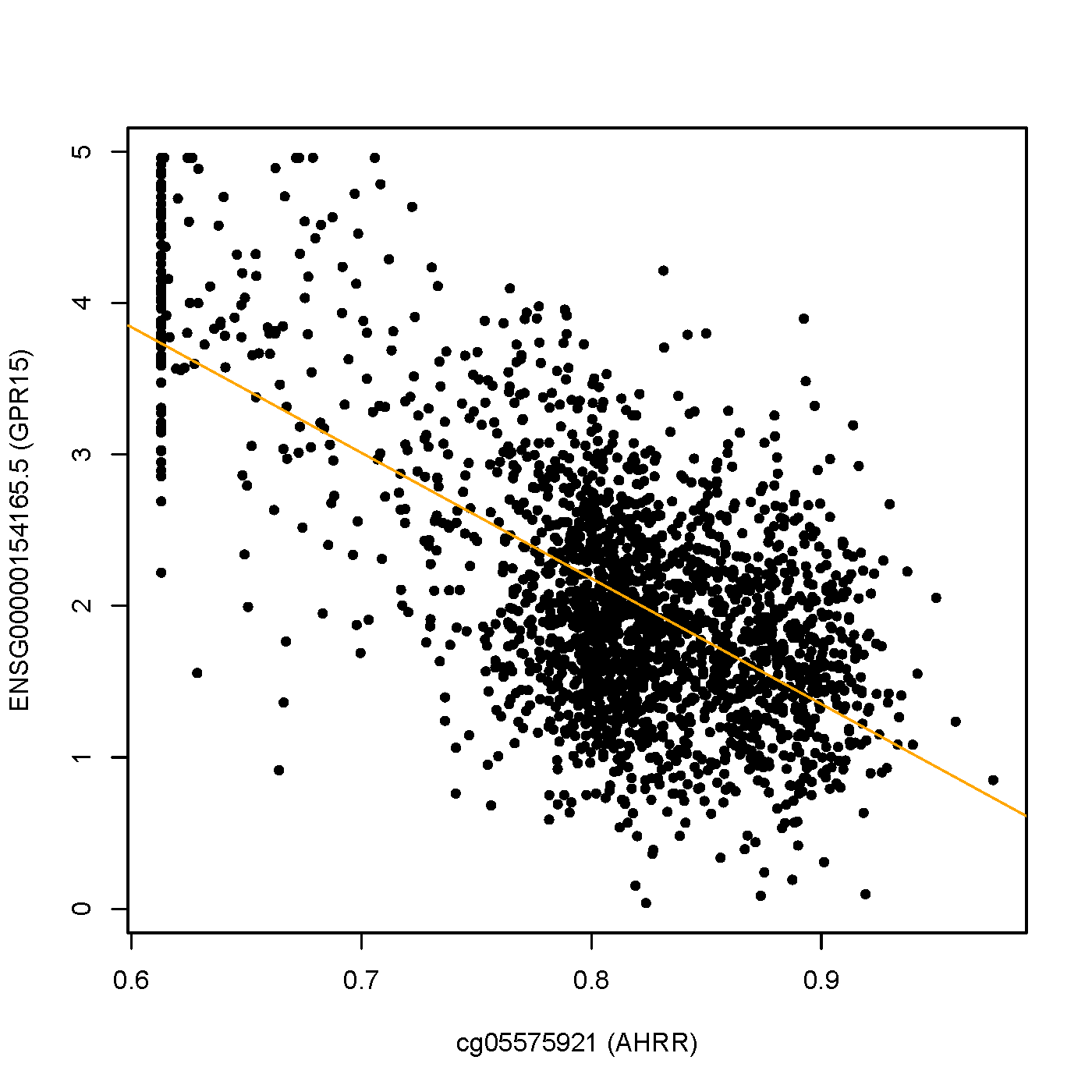


Supplemental Figure 11. *Trans* association between DNA methylation at cg13704117 and expression of ENSG00000214425.7.


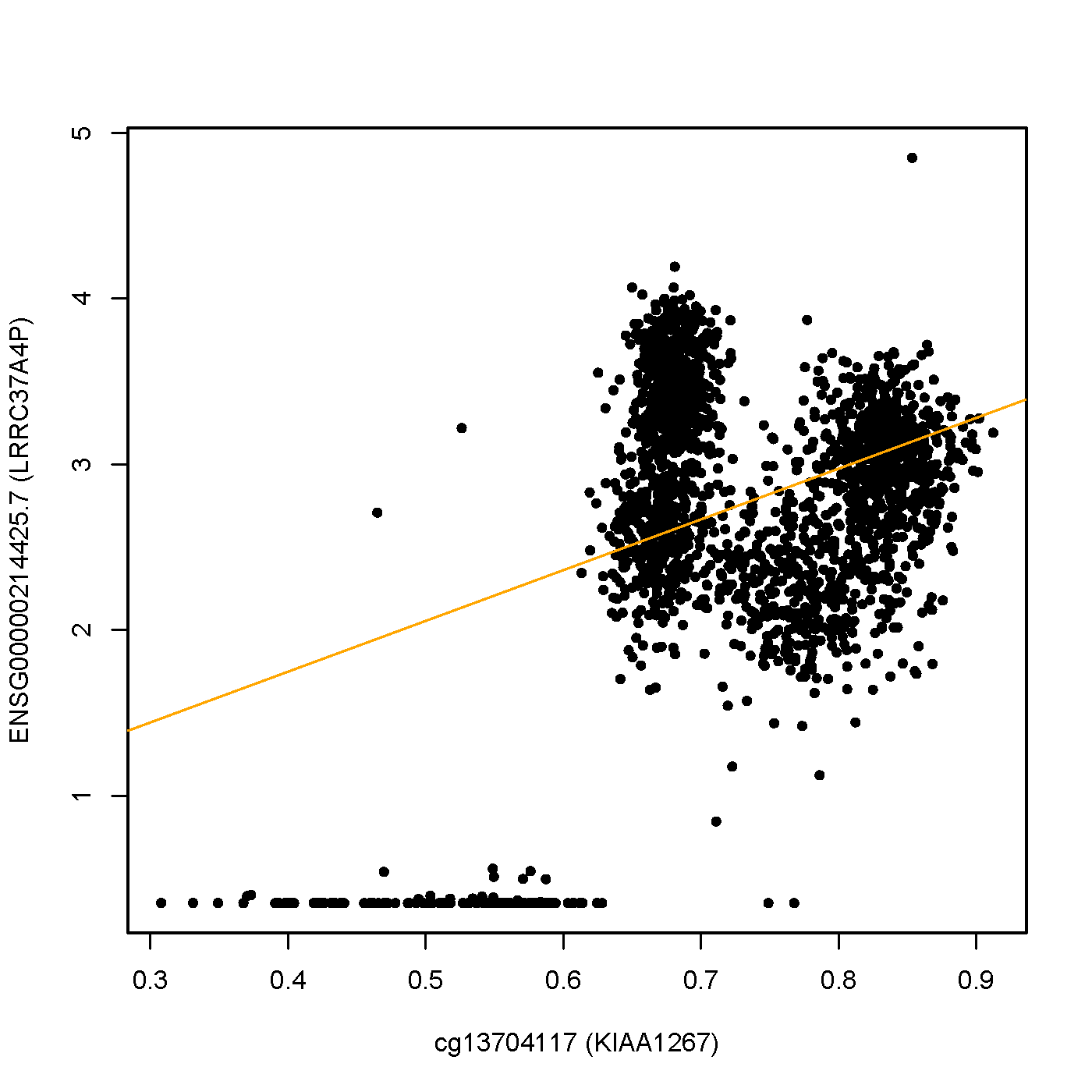

Supplement: Supplementary file 2 — Supplementary Figures. [file 41598_2023_39936_MOESM2_ESM.docx]
